# Activation of the Interleukin-33/ST2 Pathway Exerts Deleterious Effects in Myxomatous Mitral Valve Disease

**DOI:** 10.3390/ijms22052310

**Published:** 2021-02-25

**Authors:** Amaia Garcia-Pena, Jaime Ibarrola, Adela Navarro, Alba Sadaba, Carolina Tiraplegui, Mattie Garaikoetxea, Vanessa Arrieta, Lara Matilla, Amaya Fernández-Celis, Rafael Sadaba, Virginia Alvarez, Alicia Gainza, Eva Jover, Natalia López-Andrés

**Affiliations:** Cardiovascular Translational Research, Navarrabiomed (Miguel Servet Foundation), Complejo Hospitalario de Navarra (CHN), Universidad Pública de Navarra (UPNA), IdiSNA. Irunlarrea 3, 31008 Pamplona, Spain; pena.urtasun@navarra.es (A.G.-P.); jaime.ibarrola.ulzurrun@navarra.es (J.I.); anavarroe@alumni.unav.es (A.N.); alba.sadaba.cipriain@navarra.es (A.S.); carolina.tiraplegui.garjon@navarra.es (C.T.); mattiegaraiko@gmail.com (M.G.); vanessa.arrieta.paniagua@navarra.es (V.A.); lara.matilla.cuenca@navarra.es (L.M.); amaya.fernandez.decelis@navarra.es (A.F.-C.); jr.sadaba.sagredo@navarra.es (R.S.); virginia.alvarez.asiain@navarra.es (V.A.); alicia.gainza.calleja@navarra.es (A.G.); eva.jover.garcia@navarra.es (E.J.)

**Keywords:** interleukin-33, ST2, mitral valve, myxomatous, valve endothelial cells, valve interstitial cells

## Abstract

Mitral valve disease (MVD) is a frequent cause of heart failure and death worldwide, but its etiopathogenesis is not fully understood. Interleukin (IL)-33 regulates inflammation and thrombosis in the vascular endothelium and may play a role in the atherosclerotic process, but its role in mitral valve has not been investigated. We aim to explore IL-33 as a possible inductor of myxomatous degeneration in human mitral valves. We enrolled 103 patients suffering from severe mitral regurgitation due to myxomatous degeneration undergoing mitral valve replacement. Immunohistochemistry of the resected leaflets showed IL-33 and ST2 expression in both valve interstitial cells (VICs) and valve endothelial cells (VECs). Positive correlations were found between the levels of IL-33 and molecules implicated in the development of myxomatous MVD, such as proteoglycans, extracellular matrix remodeling enzymes (matrix metalloproteinases and their tissue inhibitors), inflammatory and fibrotic markers. Stimulation of single cell cultures of VICs and VECs with recombinant human IL-33 induced the expression of activated VIC markers, endothelial–mesenchymal transition of VECs, proteoglycan synthesis, inflammatory molecules and extracellular matrix turnover. Our findings suggest that the IL-33/ST2 system may be involved in the development of myxomatous MVD by enhancing extracellular matrix remodeling.

## 1. Introduction

Mitral valve disease (MVD) is the most common valvular heart condition, with an estimated prevalence of 1.8% moderate or severe MVD (either mitral regurgitation or stenosis) in the general population [1]. MVD is a major source of morbidity and death worldwide and a frequent cause of heart failure [2]. The etiology of MVD can be broadly divided into primary and secondary based on whether the mitral leaflets exhibit significant pathological abnormalities or not [3]. The commonest cause of primary MVD is myxomatous degeneration, most frequently mitral valve prolapse (MVP) [3,4]. The histopathology is characterized by architectural changes in the leaflets consisting of fragmentation of collagen with an increase in extracellular proteoglycans (PGs) in the spongiosa layer of the leaflet [5,6]. The main cellular components of cardiac valves are the valve endothelial cells (VECs) and valve interstitial cells (VICs), which maintain tissue homeostasis [7,8]. In myxomatous valves, VICs exhibit features of activated myofibroblasts and express excessive levels of PGs and proteolytic enzymes such as matrix metalloproteinases (MMPs), suggesting a main role of this cell type in the development of myxomatous degeneration [9]. Another mechanism implicated in mitral valve disease is the differentiation of VECs into an activated interstitial cell phenotype, through a process termed endothelial–mesenchymal transition (EndMT) [10,11], which leads to a high proliferation of the activated VICs and consequently to a loss of tissue homeostasis.

Interleukin (IL)-33 is a cytokine belonging to the IL-1 family that acts as an “alarmin” or stress response cytokine binding to its receptor ST2 [12]. Two distinct isoforms, ST2L (the transmembrane form) and sST2 (the soluble form), are produced by alternative splicing of the ST2 gene [13]. The interaction between IL-33 and ST2L was initially found to be cardioprotective in experimental models by reducing myocardial fibrosis, preventing cardiomyocyte hypertrophy, reducing apoptosis and improving myocardial function [13,14,15,16]. sST2 avidly binds IL-33, preventing its interaction with ST2L and thus is considered as a decoy receptor [15]. However, in the recent years deleterious consequences of the IL-33/ST2L interaction have been described [17,18]. IL-33 has been found to be a potent modulator of vascular endothelial cell activation by promoting leukocyte adhesion and proinflammatory cytokine expression [19,20]. Moreover, a procoagulant effect of IL-33 by inducing the expression of tissue factor has also been described [21].

Regarding the cardiac valves, the expression of IL-33 and ST2 has been reported in aortic valves [22] and recently a role of IL-33 in the progression of nonrheumatic aortic valve stenosis has been described via VIC differentiation into myofibroblasts and osteoblasts [23].

However, the possible role of the IL-33/ST2 in mitral valve disease has not been elucidated. In the present study, we hypothesize that the activation of the IL-33/ST2 pathway could induce myxomatous mitral valve degeneration. To test this hypothesis, we analyzed human mitral valves from patients suffering from myxomatous MVD undergoing mitral valve replacement. Our results show that leaflet levels of IL-33 correlate with histological features of myxomatous degeneration and also that in vitro stimulation of VICs and VECs with IL-33 induced the synthesis of molecules involved in the development of myxomatous MVD.

## 2. Results

### 2.1. Clinicopathological Features of the Patients with MVD

Clinicopathological features of the patients included are shown in Table 1. We included 103 patients and 65% were male, consistent with other surgical cohorts [24]. A significant proportion suffered from concomitant hypertension, hyperlipidemia and atrial fibrillation, with a prevalence similar to the aforementioned cohorts [24]. Echocardiographic variables were those expected in patients with severe mitral regurgitation with indication for surgical intervention.

### 2.2. Distribution of the IL-33 and ST2 within the Mitral Valve

**Figure 1:**
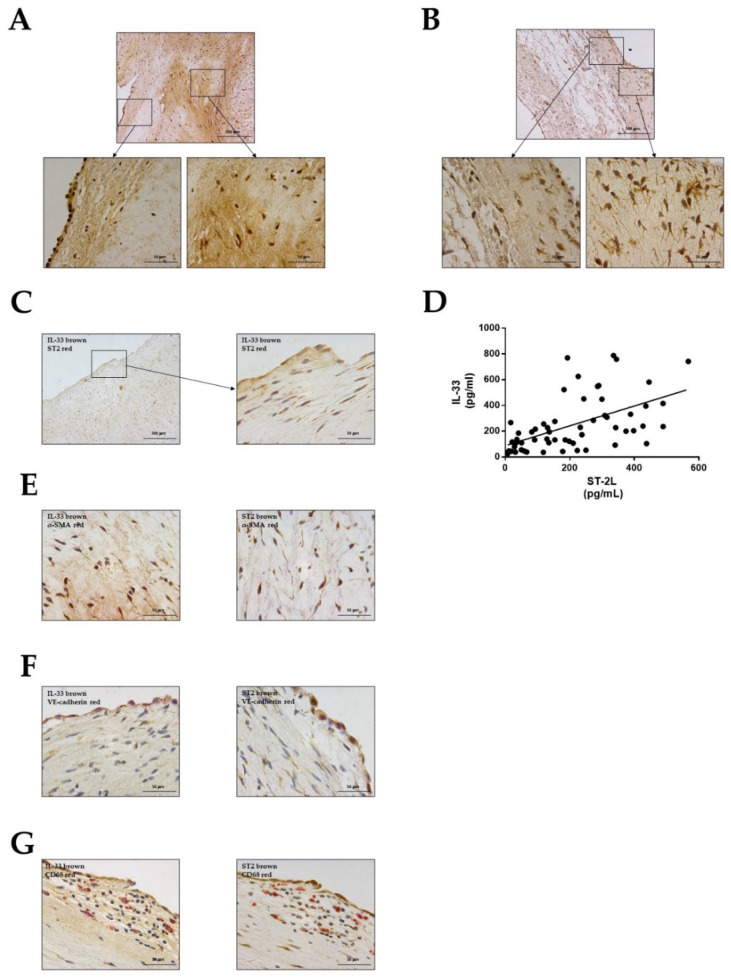
IL-33 and ST2 expression in myxomatous mitral valves. Representative microphotographs at low and high magnification of human myxomatous mitral valve sections stained for IL-33 (**A**), ST-2 (**B**) and double immunohistochemistry for IL-33 (brown) and ST-2 (red) (**C**). Correlation between IL-33 and ST-2 (*r* = 0.548, *p* < 0.0001) protein levels in human myxomatous mitral valves (**D**). Representative microphotographs of immunohistochemistry double staining for IL-33 (brown) and α-SMA (red) on the left and ST2 (brown) and α-SMA (red) on the right (**E**), IL-33 (brown) and VE-cadherin (red) (left) and ST2 (brown) and VE-cadherin (red) (right) (**F**) and IL-33 (brown) and CD68 (red) (left) and ST2 (brown) and CD68 (red) (right) (**G**). Magnification 50× (Scale bar 500 µm) and 400× (Scale bar 50 µm). Protein data in pg/mL. IL-33: Interleukin-33; α-SMA: Alpha-smooth muscle actin.

To explore the possible role of IL-33/ST2 system in myxomatous MVD, immunohistochemical analyses were performed. Myxomatous mitral valves showed that both IL-33 (Figure 1A) and ST2 (Figure 1B) were expressed in the leaflet and frequently co-localize in the same cells (Figure 1C). Regarding cellular localization, we observed that IL-33 is found predominantly in the nucleus and the expression of ST2 is mainly cytoplasmic (Figure 1A–C). Besides, a positive correlation in protein levels of IL-33 and ST2L in myxomatous mitral valves (*r* = 0.548, *p* < 0.0001) was confirmed (Figure 1D). Immunohistochemistry also revealed co-expression of both IL-33 and ST2 with α-SMA (Figure 1E) and with VE-cadherin (Figure 1F), confirming that both molecules are expressed in VICs and VECs, respectively. Immunostaining for CD68 showed scarce macrophagic infiltration adjacent to the endothelium but without significant co-expression of IL-33 nor ST2 (Figure 1G).

### 2.3. Correlation of IL-33 in Myxomatous MVs with Inflammatory Mediators, Extracellular Matrix Components, and Matrix Metalloproteinases

**Figure 2:**
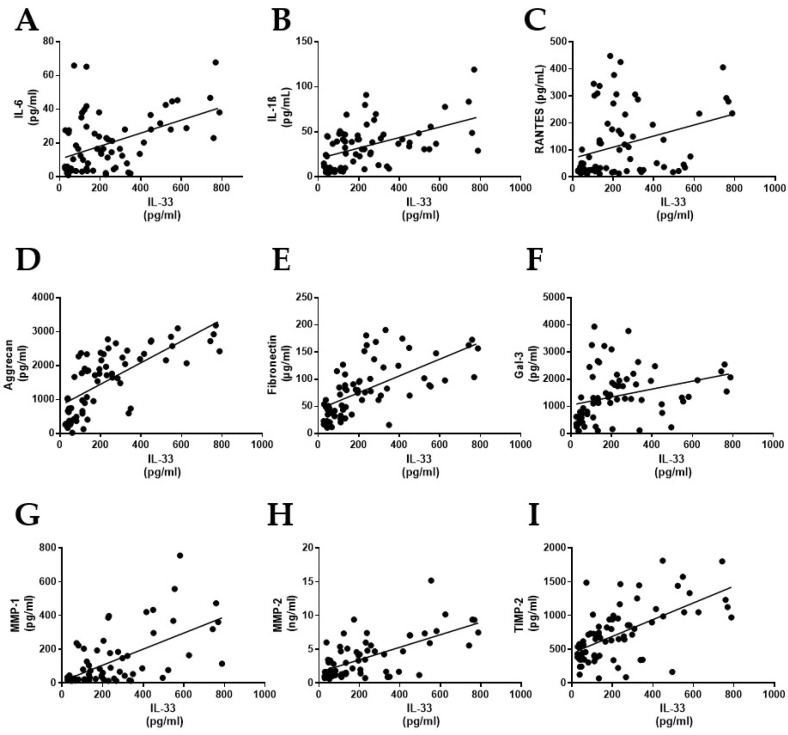
Correlations between IL-33 protein levels and principal features that characterize human myxomatous mitral valves. Correlation between valve tissue levels of IL-33 and IL-6 (*r* = 0.468, *p* < 0.0001) (**A**), IL-1β (*r* = 0.496, *p* < 0.0001) (**B**), RANTES (*r* = 0.331, *p* = 0.0037) (**C**), aggrecan (*r* = 0.711, *p* < 0.0001) (**D**), fibronectin (*r* = 0.634, *p* < 0.0001) (**E**), Gal-3 (*r* = 0.314, *p* < 0.0076) (**F**), MMP-1 (*r* = 0.618, *p* < 0.0001) (**G**), MMP-2 (*r* = 0.638, *p* < 0.0001) (**H**) and TIMP-2 (*r* = 0.606, *p* < 0.0001) (**I**). Protein data in pg/mL. IL-33: Interleukin-33; IL-6: Interleukin-6; IL-1β: Interleukin-1 beta; Gal-3: Galectin-3; MMP-1: Matrix metalloproteinase-1; MMP-2: Matrix metalloproteinase-2: TIMP-2: Tissue inhibitor of metalloproteinase-2.

To investigate the possible association between IL-33 protein levels and the molecules involved in the development of myxomatous mitral valve degeneration, we measured protein levels in the tissue homogenate by means of ELISA. Afterwards, correlation analyses were performed. The association analysis revealed moderate positive correlation of IL-33 protein levels with inflammatory markers IL-6 (*r* = 0.468, *p* < 0.0001) (Figure 2A), IL-1β (*r* = 0.496, *p* < 0.0001) (Figure 2B) and RANTES (*r* = 0.331, *p* = 0.0037) (Figure 2C). Regarding extracellular matrix components, a strong positive correlation was demonstrated between IL-33 levels and the PG aggrecan (*r* = 0.711, *p* < 0.0001) (Figure 2D) and a moderate correlation with fibronectin levels (*r* = 0.634, *p* < 0.0001) (Figure 2E). A correlation between IL-33 levels and the profibrotic marker Gal-3 (*r* = 0.314, *p* < 0.0076) was shown (Figure 2F). In relation to ECM turnover, a positive correlation was found between valve IL-33 levels and MMP-1 (*r* = 0.618, *p* < 0.0001) (Figure 2G), MMP-2 (*r* = 0.638, *p* < 0.0001) (Figure 2H) and TIMP-2 (*r* = 0.606, *p* < 0.0001) (Figure 2I).

### 2.4. IL-33 Induces Activation of Human Mitral VICs, Inflammation, Proteoglycans Synthesis and Extracellular Matrix Remodeling

**Figure 3:**
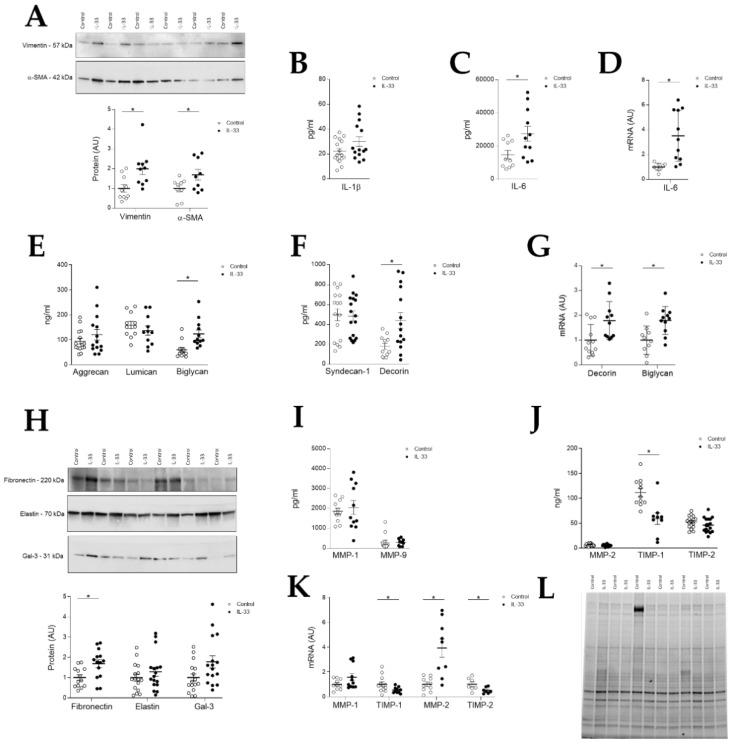
Effects of IL-33 on human mitral VICs. (**A**) Quantification of VICs activation markers (vimentin and α-SMA) in VICs treated with IL-33; (**B**,**C**) Quantification of secreted inflammatory markers (IL-1β and IL-6) in VICs treated with IL-33; (**D**) Quantification of mRNA of IL-6 in VICs treated with IL-33; (**E**) Quantification of extracellular proteoglycans aggrecan, lumican and biglycan in VICs treated with IL-33; (**F**) Quantification of secreted proteoglycans syndecan-1 and decorin in VICs treated with IL-33; (**G**) Quantification of mRNA of decorin and biglycan in VICs treated with IL-33; (**H**) Quantification of fibronectin, elastin and Galectin-3 at protein levels in VICs treated with IL-33; (**I**) Quantification of secreted MMP-1 and MMP-9, (**J**) TIMP-1, MMP-2, TIMP-2 at protein levels in VICs treated with IL-33; (**K**) Quantification of mRNA of MMP-1, TIMP-1, MMP-2 and TIMP-2 in VICs treated with IL-33; (**L**) Representative Stain free. Box plots show the individual datapoints and the horizontal bars indicate the mean and SEM in arbitrary units versus the control group. For Western blot experiments, protein densitometry was expressed in arbitrary units (AU) once normalized to stain-free and β-actin. Representative blots have been displayed when appropriate. * *p* < 0.05 vs. control. IL-33: α-SMA: Alpha-smooth muscle actin; IL: Interleukin; Gal-3: Galectin-3; MMP: Matrix metalloproteinase; TIMP: Tissue inhibitor of metalloproteinase.

After treatment with human recombinant IL-33, VICs isolated from myxomatous mitral valves showed a significant increase in the expression of the markers of activated VICs vimentin (*p* = 0.0052) and α-SMA (*p* = 0.0444) (Figure 3A). IL-33 administration also induced a significant increase in the secretion of inflammatory molecules such as IL-6 (*p* = 0.0356), with a slight increase in IL-1β protein levels that did not reach statistical significance (Figure 3B). Moreover, IL-6 was also increased by IL-33 treatment at the mRNA and the protein levels (Figure 3C,D).

To determine if IL-33 induces PGs synthesis, a major feature of myxomatous mitral valve degeneration, we assessed the extracellular protein levels of aggrecan, lumican, syndecan-1, decorin and biglycan (Figure 3E–G). Only biglycan (*p* = 0.0012) and decorin (*p* = 0.0117) exhibited a significant increase both at protein (Figure 3E,F) and mRNA levels (Figure 3G). IL-33 treated cells also presented a significant increase in other non-fibrillar proteins such as fibronectin (*p* = 0.0082) (Figure 3H). Levels of other ECM components, like elastin, were not affected by the administration of IL-33 (Figure 3H). The profibrotic marker Gal-3 presented a non-significant increase after IL-33 stimulation (Figure 3H).

Regarding ECM turnover, stimulation with IL-33 did not modify the secretion of MMP-1, MMP-9 (Figure 3I), MMP-2 or TIMP-2 (Figure 3J). However, treatment with IL-33 significantly decreased the secretion of TIMP-1 (*p* = 0.0013) (Figure 3J). At the mRNA level, TIMP-1 and -2 secretion was reduced by IL-33 (*p* = 0.0444 for TIMP-1 and *p* = 0.0393 for TIMP-2) (Figure 3K). IL-33-treated cells also exhibited significantly higher levels of MMP-2 at mRNA levels (*p* = 0.0009) (Figure 3K).

See stain free for original Western blot images in Figure 3L.

### 2.5. IL-33 Induces EndMT in Valve Endothelial Cells and Extracellular Matrix Changes

**Figure 4:**
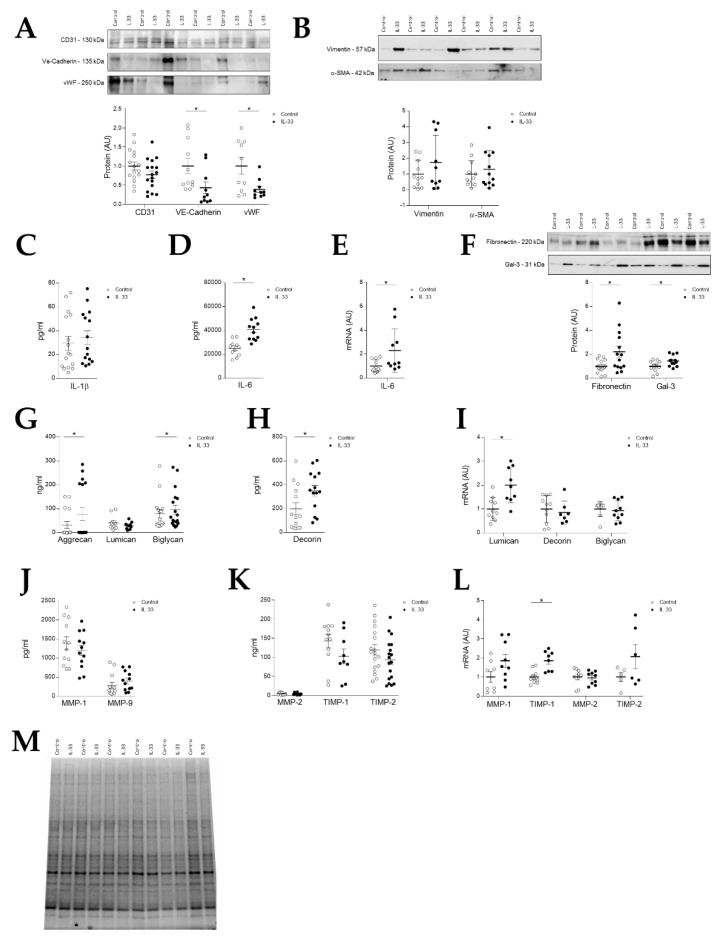
Effects of IL-33 on human mitral VECs. (**A**) Quantification of VECs markers (CD31, VE-cadherin and Von Willebrand factor) in VECs treated with IL-33 at protein levels. (**B**) Quantification of VICs activation markers (vimentin and α-SMA) in VECs treated with IL-33 at protein levels. (**C**,**D**) Quantification of secreted inflammatory markers IL-1β and IL-6 in VECs treated with IL-33. (**E**) Quantification of mRNA of IL-6 in VICs treated with IL-33; (**F**) Quantification of fibronectin and Galectin-3 at protein levels in VICs treated with IL-33; (**G**) Quantification of extracellular proteoglycans aggrecan, lumican and biglycan in VECs treated with IL-33; (**H**) Quantification of secreted decorin in VECs treated with IL-33; (**I**) Quantification of mRNA of lumican, decorin and biglycan in VECs treated with IL-33; (**J**) Quantification of secreted MMP-1 and MMP-9, (**K**) TIMP-1, MMP-2, TIMP-2 at protein levels in VECs treated with IL-33; (**L**) Quantification of mRNA of MMP-1, TIMP-1, MMP-2 and TIMP-2 in VECs treated with IL-33; (**M**) Representative Stain free. Box plots show the individual datapoints and the horizontal bars indicate the mean and SEM in arbitrary units versus the control group. For Western blot experiments, protein densitometry was expressed in arbitrary units (AU) once normalized to stain-free and b-actin. Representative blots have been displayed when appropriate. * *p* < 0.05 vs. control. IL: Interleukin; vWF: Von Willebrand factor; Gal-3: Galectin-3; MMP: Matrix metalloproteinase; TIMP: Tissue inhibitor of metalloproteinase.

After treating VECs with IL-33, we observed a diminished expression of valve endothelial cell markers such as VE-cadherin (*p* = 0.0127) and von Willebrand Factor (*p* = 0.0288), a feature of EndMT (Figure 4A). There was also a parallel increase in the expression of activated VICs, like vimentin and α-SMA, though not significant (Figure 4B).

Treatment of VECs with IL-33 induced a similar pattern of the synthesis of inflammation markers compared to IL-33-treated VICs. Whereas IL-1β secretion was not affected by IL-33 treatment (Figure 4C), a significant increase in both IL-6 secreted protein (*p* = 0.0001) (Figure 4D) and mRNA (*p* = 0.0429) (Figure 4E) levels was observed. IL-33 also augmented the protein expression of fibronectin (*p* = 0.0149) and Gal-3 (*p* = 0.0290) (Figure 4F).

Regarding PGs, IL-33 treated VECs exhibited significantly higher protein levels of aggrecan (*p* = 0.0215) with similar levels of other PGs as compared to controls (Figure 4G). Secreted decorin was increased by IL-33 treatment (*p* = 0.0309) in VECs (Figure 4H). IL-33-treated cells also exhibited significantly higher levels of lumican at mRNA levels (*p* = 0.0025) (Figure 4I).

Levels of secreted MMPs and TIMPs did not differ between IL-33-treated VECs and controls, neither at protein (Figure 4J,K). Interestingly, stimulation of VECs with IL-33 induced an increase in the mRNA levels of TIMP-1 (*p* = 0.0007) (Figure 4L). 

See stain free for original Western blot images in Figure 4M.

## 3. Discussion

The present study is the first one to assess the involvement of the IL-33/ST2 pathway in the development of mitral valve disease. We have demonstrated for the first time the expression of IL-33 and ST2L in mitral valves and their colocalization with VICs and VECs. Moreover, we found that the tissue levels of IL-33 correlate with the levels of molecules implicated in the development of myxomatous degeneration of the mitral valve, such as inflammatory cytokines and extracellular matrix components and matrix metalloproteinases. In in vitro experiments stimulating human mitral VICs and VECs from patients with myxomatous MVD with recombinant IL-33, we have demonstrated that it induces activation of VICs, EndMT of VECs and extracellular matrix remodeling. All of these have been previously identified as key components of myxomatous MVD development [9,10,11].

The expression of IL-33 and ST2 in cardiac valves was explored for the first time in 2012 by Sawada et al. in aortic valves [22]. The majority of IL-33 positive cells were CD68 positive macrophages although IL-33 was also expressed by VICs and VECs. ST2 was expressed predominantly by CD68 positive macrophages [22]. Our results show that in mitral valves IL-33 was predominantly found in VICs and VECs, more specifically in the nucleus, consistent with previous studies [20]. Interestingly, we observed a significant presence of ST2 in the cytoplasm of VICs and VECs. These findings were further supported by a positive correlation between protein levels of IL33 and ST2L.

Histologically, myxomatous valves are characterized by an expansion of the spongiosa layer due to the accumulation of extracellular PGs. More specifically, it has been shown that the PGs decorin, biglycan and versican are significantly increased when compared to normal mitral valves [6,9]. Interestingly, in our study, stimulation of both VICs and VECs with IL-33 exerted a significant increase in decorin and biglycan synthesis. Stimulation of VECs also induced a significant increase in the non-fibrillar PG aggrecan, in line with the strong positive correlation observed between the tissue levels of this PG and IL-33. Aggrecan is a hyalectan that confers viscoelasticity and load-bearing properties to tissues in which it is expressed [25], and its accumulation has been described in myxomatous valves of a murine model of Marfan syndrome [26]. Another key feature of myxomatous MVD is an overall increased expression of MMPs [19]. In line with this, we observed that the leaflet levels of IL-33 correlated with the levels of MMP-1 and -2 and their tissue inhibitors -1 and 2. Moreover, we found positive correlations of IL-33 levels with the proinflammatory cytokine IL-1β, which is known to induce the expression of MMPs in VICs [19]. These results suggested the direct implication of IL-33 in the production and remodeling of mitral valve extracellular matrix.

Two cellular processes have been described in the development of MVD: aberrant activation of quiescent VICs [9] and endothelial–mesenchymal transition [10,11] leading to proliferation of activated VICs. Vimentin and α-SMA, two molecules that characterize the phenotype of activated VICs that precedes PGs synthesis found in myxomatous valves [9], increased after stimulation of VICs with IL-33. On the other hand, VECs treated with IL-33 presented a significant loss of VEC markers, suggesting a possible role of IL-33 in the induction of EndMT of VECs. This is in line with the work of He et al. that explored the role of IL-33 in aortic valve stenosis [23]. They observed that stimulation of VICs from porcine aortic valves with IL-33 induced the expression of α-SMA, as well as calcification markers. In the present work we demonstrated that IL-33 directly induced the activation of VICs and VECs, contributing to the myxomatous mitral valve phenotype.

We also observed positive correlations between the tissue levels of IL-33 and IL-6, an interleukin that has been described as an activator of aortic VICs [27] and an inductor of EndMT in VECs [28]. Moreover, when stimulated with IL-33, both VICs and VECs exhibited a dramatic increase in the synthesis of IL-6, suggesting that it may also be involved in the development of MVD, in the same way as described in aortic VICs [27] and VECs [28]. Recent studies have shown that the treatment of murine cardiac fibroblasts with IL-33 yielded a dose-dependent increase in the expression of IL-6 and monocyte chemotactic protein-1 (MCP-1) [29]. Moreover, it has also been demonstrated a role of IL-33 in vascular endothelial cell activation by promoting adhesion molecules and proinflammatory cytokine expression, respectively MCP-1 and IL-6, among others [19]. In the present study, we have shown that both VICs and VECs are a target for IL-33-related induction of myxomatous degeneration features.

On the other hand, we found a much lesser involvement of inflammatory cells than reported in aortic valves [22]. Although some degree of lymphocytes and macrophages infiltration in non-rheumatic regurgitant mitral valves has been described, it is significantly lower than in calcific aortic stenosis [30,31], suggesting differential pathophysiological mechanisms with a more prominent role of VICs and VECs in mitral valve disease than in calcific aortic degeneration -which presents features more similar to the atherosclerotic process. The prevalence in our patients of coronary artery disease, defined as coronary stenosis ≥50% of the lumen, was 21%, mostly subclinical, since only 6% had previously had myocardial infarction. These rates are lower than reported in epidemiological studies, which report a myocardial infarction prevalence in men aged 60 to 79 years (which comprises the majority of our patients) of 11.3%, and 4.2% in women of the same age [32]. Moreover, the circulating CRP levels were within normal range. In summary, these findings suggest that inflammation may play a minor role in the pathogeny of MVD, and the effects of the inflammatory cytokines such as IL-6 may be mediated via VICs activation or VECs EndMT rather than implicating the participation of cells of the immune system.

This study comprises several limitations: first, we did not have access to non-diseased mitral valves as controls due to the difficulty of obtaining informed consent at autopsies. Second, VICs and VECs were obtained from individuals suffering from advanced stages of the disease requiring surgery, so the results may not be extrapolated to initial stages of the disease when preventing the progression of the disease would be critical. Third, VICs and VECs were isolated from human valves from MVP patients of similar age with similar risk factors, although the cells were not separated by individual or sex. However, this is an initial and exploratory study that opens a new field of research in mitral valve pathogeny.

In summary, this study has identified IL-33 as a new pathway involved in the development of myxomatous mitral valve disease. Based on our results, we consider that IL-33 activates quiescent VICs, induces endothelial–mesenchymal transition of VECs and also induces the synthesis of extracellular matrix components that may ultimately lead to the development of myxomatous degeneration of the valve. Unravelling the physiopathology of MVD is key to develop new targeted therapies for preventing disease progression. In spite of the high prevalence of MVD, currently no pharmacological treatment has been identified and, accordingly, is the second most-frequent indication for valve surgery in Europe [33]. MVD has a significant prognostic impact even with surgical treatment, with a 10-year survival rate of 86% when early surgery is performed and 69% survival rate when watchful waiting strategy is adopted [34]. For these reasons, identifying novel druggable targets if of utmost importance.

## 4. Materials and Methods

### 4.1. Patient Population

This prospective, observational study included 103 consecutive cases of patients undergoing mitral valve replacement in our institution, from June 2015 to November 2018, due to significant MVP caused by myxomatous valve disease not amenable to surgical repair. MVP was defined by transthoracic echocardiography if there was systolic displacement of the mitral leaflet into the left atrium at least 2 mm from the mitral annular plane in the parasternal long axis window, following current guidelines [3]. Exclusion criteria were other etiologies of MVD such as rheumatic, senile degeneration, infectious, ischemic, etc. Patients with dysfunction of other heart valves were not excluded.

All patients underwent transthoracic echocardiogram evaluation and in 63% transesophageal echocardiography was performed based on clinician’s criterion. Peripheral blood samples were extracted within the 24 h prior to the surgery.

Informed consent was obtained from each patient and the study protocol conforms to the ethical guidelines of the 1975 Declaration of Helsinki as reflected in a priori approval by the institution’s human research committee (project 26/2013).

### 4.2. Immunohistological Evaluation

Histological determinations in mitral valves were performed in 5 μm thick sections of paraffin-embedded human myxomatous mitral valves. The immunochemistry was performed following the protocol of Leica BOND-Polymer Refine Detection automatic immunostainer (Leica Biosystems, Wetzlar, Germany). All solutions were filled into the bottle, Bond Open Container (Leica), and registered on a computer using the Leica Biosystem program. The immunostaining program protocol include: Fixative solution, Bond wash solution, Blocking with common immunohistochemistry blocker and incubated with the primary antibody for IL-33 (Santa Cruz Biotechnology, CA, USA), ST2 (Abcam, Cambridge, UK), α-SMA (Sigma-Aldrich, Sigma/Merck Life Sciences S.L.U., Madrid, Spain), VE-cadherin (Santa Cruz Biotechnology) and CD-68 (Santa Cruz Biotechnology). After primary antibody incubation, slides were incubated with post primary poly-HRP-IgG. The signal was revealed by using DAB Substrate. As negative controls, samples followed the same procedure described above but in the absence of primary antibodies were used. For each immunochemistry and staining, serial sections were done. In the figures the most representative image is shown.

### 4.3. ELISA

IL-33, IL-6, IL-1β, RANTES, aggrecan, lumican, syndecan-1, decorin, fibronectin, Gal-3, MMP-1, TIMP-1, MMP-2, TIMP-2, MMP-9 were measured in valve extracts and cell supernatants by ELISA according to the manufacturer’s instructions (R&D Systems, Minneapolis, MN, USA). ST-2 expression was quantified in valve extracts using the Critical Diagnostics ELISA kit. Biglycan expression was measured by ELISA following the manufacturer’s instructions (Sigma-Aldrich).

### 4.4. Cell Isolation and Culture

Human VICs and VECs were isolated from 7–9 human mitral valves obtained from mitral valve replacement surgery. Mitral valves were incubated with Collagenase type 2 (240 U/mg) (Worthington Biochemical Corporation, Freehold, NJ) for 10 min four times for VECs isolation and for 1 h one time for VICs isolation. For VECs isolation, supernatants were re-collected and centrifuge 5 min at 10,000× *g*. VECs were grown on 2% gelatin-coated dishes in EBM-2 medium supplemented with GA1000, Hydrocortisone solution, Fetal Bovine Serum, Human Fibroblast Growth Factor basic (hFGFb), Human Vascular Endothelial Growth Factor (hVEGF), Analog of Human Insulin-Like Growth Factor-1, Long R3-IGF-1, Ascorbic Acid Solution and Human Epidermal (Lonza, Basel, Switzerland). For VICs, after 1 h in Collagenase type 2 (240 U/mg), supernatant was centrifuged 5 min at 10,000× *g* VICs were cultured on DMEM F-12 (Gibco, Invitrogen, Ghent, Belgium) medium supplemented with 10% FBS. All assays were done at 37 °C, 95% sterile air and 5% CO_2_ in a saturation humidified incubator. The VIC phenotype of isolated cells was confirmed through immunocytochemistry by probing for the VIC markers vimentin and alpha-smooth muscle actin (-SMA), whereas the VEC phenotype was confirmed by the expression of the endothelial markers CD31 and VE-cadherin (data not shown). Only cell cultures characterized by purity greater than 90% were used for subsequent studies. Cells were used between passages 3–4 for VECs and 3–6 for VICs.

### 4.5. Single Culture Cell Assays

VICs were treated with IL-33 at concentration of 20 ng/mL for 6 h for mRNA studies and for 24 h for protein studies. VECs were treated with IL-33 at doses of 20 ng/mL for 24 h for mRNA studies and for 96 h for protein studies.

### 4.6. Real-Time Reverse Transcription PCR

Total RNA was extracted with Trizol Reagent (Qiagen, Hilden, Germany), according to the manufacturer’s instructions. First strand cDNA was synthesized according to the manufacturer’s instructions (Bio-Rad, Hercules, CA, USA). Quantitative PCR analysis was performed with SYBR green PCR technology (Bio-Rad). The following primers were used: IL-6 (F: AGTTCCTGCAGAAAAAGGCAAAG, R: CATTTGCCGAAGAGCCCTCA); decorin (F: CCTGATGACCGCGACTTCGAG, R: TTTGGCACTTTGTCCAGACCC); biglycan (F: TCTGTCACACCCACCTACAGC, R: AGGGGAGATCTCTTTGGGCAC); lumican (F: TGAGCTGGATCTGTCCTATAA, R: ATCTTGCAGAAGCTCTTTATG), MMP-1 (F: ACATGAGTCTTTGCCGGAGG, R: AACAAGGTTGACTTTATTCCAAACA), TIMP-1 (F: GGAATGCACAGTGTTTCCCTG, R: GGAAGCCCTTTTCAGAGCCT), MMP-2 (F: CGACCACAGCCAACTACGAT, R: GTCAGGAGAGGCCCCATAGA), TIMP-2 (F: GCTGCGAGTGCAAGATCACG, R: AGAGCTGGACCAGTCGAAAC. Relative quantification was achieved with MyiQ software. Data were normalized by HPRT, GADPH and β-actin levels and expressed as percentage relative to controls. All PCRs were performed at least in triplicate for each experimental condition.

### 4.7. Western Blot Analysis

Aliquots of 20 µg of total proteins were prepared from cells and mitral valves and electrophoresed on SDS polyacrylamide gels and transferred to Hybond-c Extra nitrocellulose membranes (Bio-Rad). Membranes were incubated with primary antibodies for Vimentin (1:100, Santa Cruz Biotechnology), α-Smooth Muscle Actin (α-SMA, 1:100, Sigma-Aldrich), Fibronectin (1:100, Merck Millipore, Darmstadt, Germany), Elastin (1:50, Abcam), Gal-3 (1:100, Thermo Fisher Scientific, Waltham, Massachusetts, USA), CD31 (1:100, Abcam), vWF (1:100, Santa Cruz Biotechnology), VE-cadherin (1:100, Abcam). Stain free and β-Actin (Sigma-Aldrich) detection was used as loading control. After washing, detection was made through incubation with peroxidase-conjugated secondary antibody, and developed using an ECL chemiluminescence kit (Amersham, GE healthcare, Thermo Fisher Scientific, UK). After densitometric analyses, optical density values were expressed as arbitrary units. All Western blots were performed at least in triplicate for each experimental condition. Whole original images are displayed in the figures.

### 4.8. Statistical Analysis

For cellular studies, data were expressed as mean ± SEM. Normality of distributions was verified by means of the Kolmogorov–Smirnov test. Student’s *t*-test (2-tailed) was used to compare independent samples or conditions using GraphPad Software Inc. (San Diego, CA, USA). In each analysis, the critical significance level was set to a *p* value of <0.05.

Clinical data were expressed as mean ± SD or n (%). Correlation analyses were used to identify the relationship between mitral valve IL-33 protein levels and molecular features of myxomatous MVD. Values of *p* < 0.05 were considered statistically significant.

## Figures and Tables

**Table 1:** Clinicopathological features of the patients with mitral valve disease (MVD). Values are N = number or mean ± SD. LVEF = left ventricular ejection fraction; NYHA = New York Heart Association classification of heart failure; BNP = brain natriuretic peptide; ACE = angiotensin converting enzyme; ARB = angiotensin receptor blockers; CRP = C reactive protein; MVP = mitral valve prolapse.

Clinicopathological Features	*n*
Total number of patients	103
Age (years ± SD)	68 ± 10
Male (N, % of total)	68 (65)
Hypertension (N, % of total)	59 (56)
Hypercholesterolemia (N, % of total)	48 (46)
Diabetes mellitus (N, % of total)	9 (9)
Coronary artery disease (N, % of total)	22 (21)
Atrial fibrillation (N, % of total)	52 (50)
**Heart failure**	
NYHA functional class at the time of surgery (N, % of total)	
I	9 (9)
II	51 (49)
III	42 (40)
IV	1 (1)
Previous admission for heart failure (N, % of total)	20 (19)
BNP (pg/mL ± SD)	266 ± 342
CRP (µg/mL ± SD)	2.84 ± 1.67
**Medications**	
ACE inhibitors (N, % of total)	38 (37)
ARB (N, % of total)	21 (20)
Spironolactone (N, % of total)	6 (6)
Eplerenone (N, % of total)	3 (3)
Diuretics (N, % of total)	80 (78)
Betablockers (N, % of total)	52 (51)
Digoxin (N, % of total)	18 (17)
Statins (N, % of total)	44 (43)
**Echocardiographic parameters**	
LVEF (mean ± SD)	60.6 ± 10.4
Systolic dysfunction (LVEF <60 %) (N, % of total)	36 (35)
Severe systolic dysfunction (LVEF <35 %) (N, % of total)	2 (2)
End diastolic diameter (mm ± SD)	59 ± 7
Indexed end diastolic diameter (mm/m2 ± SD)	33 ± 4
End systolic diameter (mm ± SD)	40 ± 8
Indexed end systolic diameter (mm/m2 ± SD)	23 ± 7
Systolic pulmonary artery pressure (mmHg ± SD)	47 ± 12
**MVP etiologic subgroups**	
Barlow disease (N, % of total)	70 (68)
Fibroelastic deficiency (N, % of total)	30 (29)
Syndromic MVP	1 (1)
Associated to hypertrophic cardiomyopathy	2 (2)

## Data Availability

The data presented in this study are available on request from the corresponding author.
